# Detection of Antibodies Specific to H5 Avian Influenza Virus in a Sheep in Norway, June 2024, 11 Months After an Outbreak of Highly Pathogenic Avian Influenza in a Nearby Seabird Colony

**DOI:** 10.1111/irv.70182

**Published:** 2025-11-18

**Authors:** Johanna Hol Fosse, Grim Rømo, Francesco Bonfante, Ida Kristin Myhrvold, Kristin Stangeland Soetart, Kristin Udjus, Ragnhild Tønnessen

**Affiliations:** ^1^ Norwegian Veterinary Institute Ås Norway; ^2^ Istituto Zooprofilattico Sperimentale Delle Venezie Legnaro Italy

**Keywords:** cross‐species transmission, H5N1, highly pathogenic avian influenza, post‐outbreak surveillance, spillover infection, wildlife‐livestock interface

## Abstract

A 2023 outbreak of highly pathogenic avian influenza in seabirds in Norway caused substantial environmental contamination of grazing areas frequented by local sheep. Eleven months later, 220 sheep were tested for antibodies to type A influenza and H5 subtype using ELISA, haemagglutination inhibition and microneutralisation assays. One ewe (0.5%) tested positive by all methods, consistent with prior spillover infection. This underscores the importance of restricting livestock access to outbreak areas to mitigate cross‐species transmission and zoonotic risk.

## 1 Exposure of Grazing Sheep to Highly Pathogenic Avian Influenza Virus (HPAIV) H5N1 During an Outbreak in Seabirds in Norway, July 2023

**FIGURE 1:**
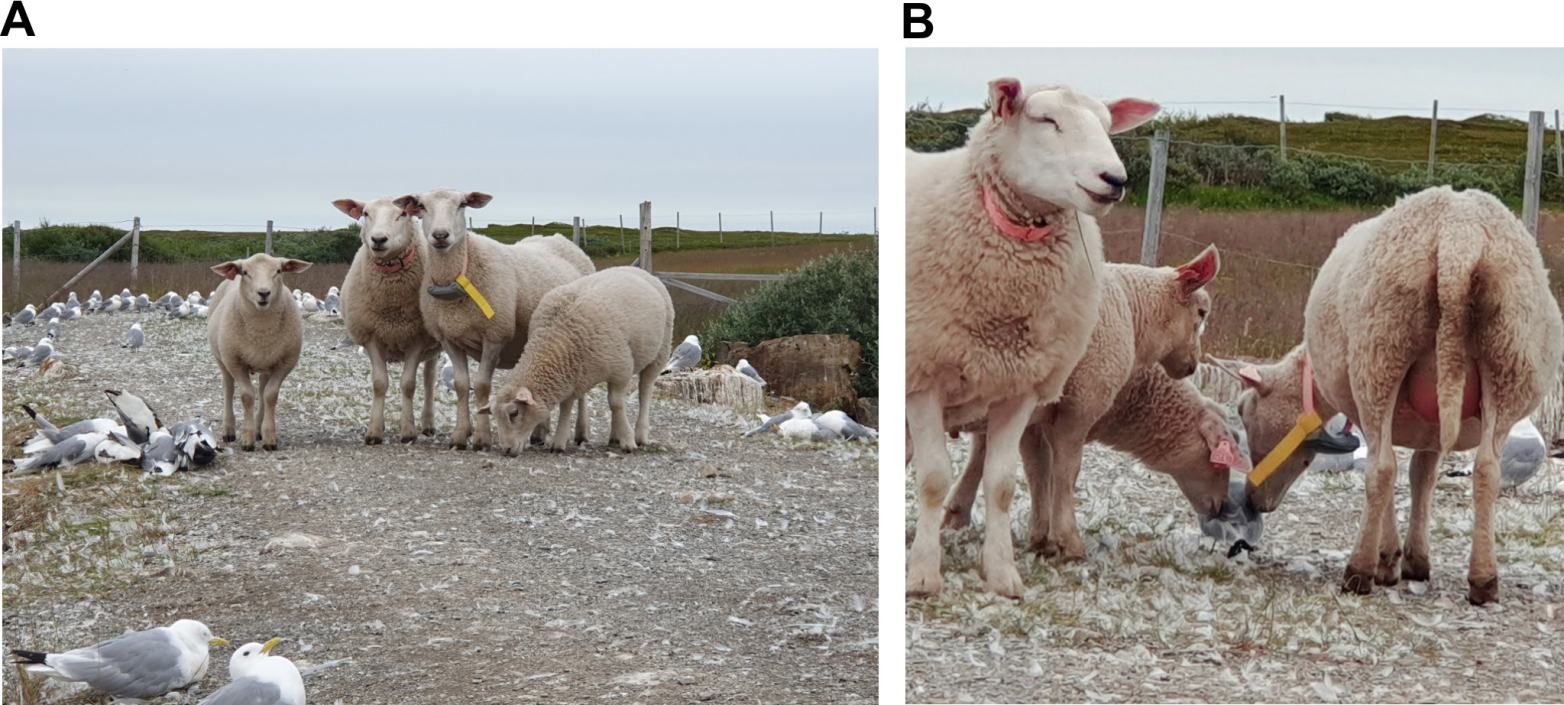
Exposure of sheep during the 2023 HPAI outbreak in seabirds. (A) Sheep surrounded by diseased and dead birds and other contaminated materials. (B) Lamb and ewe in close muzzle contact with diseased bird. Immediately after the photo was taken, the lamb fed from the ewe, illustrating a potential route for direct infection of the udder (Video S1). Photos: Grim Rømo.

Highly pathogenic avian influenza (HPAI) (H5Nx) clade 2.3.4.4b viruses have spread globally since 2020, causing fatal disease in a wide range of wild bird species with increased rates of transmission to mammals [1]. In July 2023, more than 15,000 dead seabirds were recorded in association with an outbreak caused by HPAIV H5N1 EA‐2022‐BB in northern Norway [2]. In the early phase, the number of carcasses far exceeded the capacity for removal. This resulted in substantial contamination of surrounding rough grazing areas used by local sheep (Figure 1A), including lambs observed investigating diseased birds with their muzzle before feeding from their mothers (Figure 1B and Video S1). At the time of the outbreak, ruminants were not considered susceptible to HPAI, the sheep appeared healthy and the potential for spillover infection was not investigated further.

## 2 Detection of Antibodies Specific to Type A Influenza and H5 Subtype Avian Influenza in Ovine Serum Collected in June 2024, 11 Months After HPAI Exposure

**FIGURE 2:**
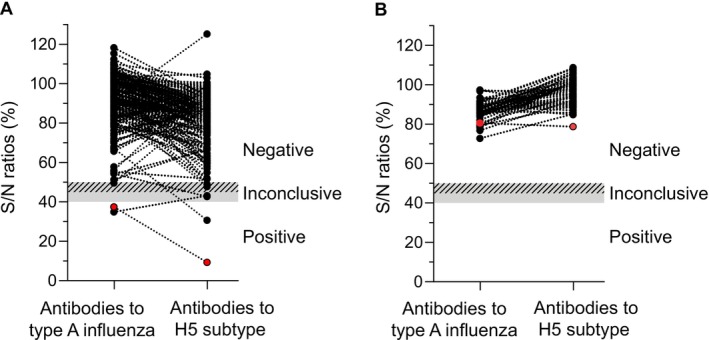
Detection of antibodies to type A influenza and H5 subtype avian influenza in sheep following HPAI exposure. (A) Serum (*n* = 220) and (B) milk (*n* = 59) samples were analysed for the presence of antibodies specific to type A influenza nucleoprotein and H5 subtype avian influenza using commercial ELISA kits (ID screen Influenza A Antibody Competition Multispecies ELISA and ID screen Influenza H5 Antibody Competition 3.0 Multispecies ELISA, both from Innovative Diagnostics, France), in accordance with the manufacturer's recommendations for bovine samples [7, 8]. Dots show individual S/N‐ratios with lines connecting results from the same sheep. The inconclusive ranges are indicated as hatched (type A influenza) and grey (H5 subtype) areas. The sheep with positive results in HI and MN serum assays is highlighted in red.

In March 2024, an extensive HPAIV H5N1 epizootic was confirmed in dairy cattle in the United States [3], raising awareness of potential spillovers from wild birds to ruminants. Clinical signs in cows were associated with mastitis, and high virus titres were detected in milk [3]. Intermammalian transmission was documented between cows, from cows to dairy farm workers, and to cats ingesting raw milk [3, 4, 5]. In June 2024, blood samples were collected from 220 Norwegian white sheep from the three farms exposed during the 2023 avian outbreak, as part of post‐outbreak surveillance. Parallel milk samples were collected from 59 lactating sheep from one farm. Serum samples were tested for the presence of antibodies specific to type A influenza nucleoprotein (anti‐NP antibodies), using a commercial ELISA (IDscreen Influenza A Antibody Competition Multi‐species ELISA, Innovative Diagnostics, France). The initial analysis, following the manufacturer's recommendations for bovine serum available at the time [6], detected no positive samples. When samples were re‐analysed in May 2025 according to updated recommendations [7], anti‐NP antibodies were detected in two ewes. One of these (#76) was born in 2022 and lambed in 2023, lactating at the time of the outbreak. This sheep also tested positive for the presence of antibodies specific to haemagglutinin subtype 5 avian influenza (anti‐H5 antibodies) in a commercial ELISA (IDscreen Influenza H5 Competition 3.0 Multispecies ELISA, Innovative Diagnostics, France), following the recommended protocol for bovine serum [8]. All sera were heat‐treated (56°C, 30 min) and analysed by haemagglutination inhibition (HI) and microneutralisation (MN) tests, using an H5 clade 2.3.4.4b virus (A/turkey/Italy/21VIR9520‐3/2021 (H5N1), genotype EA‐2020‐C). For HI, sera were also pretreated with receptor destroying enzyme (Seiken, Japan) and cross‐adsorbed with chicken red blood cells. HI was also performed against two low pathogenic avian influenza (LPAI) H5N3 viruses of Eurasian non‐Gs/Gd lineage (A/teal/England/7394/2006 and A/Mallard Duck/Netherlands/38/2008). Sheep #76 showed low‐to‐moderate neutralising activity against the H5 clade 2.3.4.4b virus (HI titres 1:10–1:20, MN titre 1:40), but did not inhibit LPAI haemagglutination, confirming a humoral response against circulating H5 clade 2.3.4.4b viruses. The other sheep (#68) that tested positive for anti‐NP antibodies was born in 2023, from a dam culled the same autumn because of mastitis. Sheep #68 tested inconclusive for anti‐H5 antibodies by ELISA and negative by both MN and HI. HI tests using antigens from relevant gull influenza viruses (A/herring gull/Norway/10_2336/2006 (H13N6) and A/herring gull/Norway/10_1623/2006 (H16N3)) also resulted negative. A third (#140) and fourth (#10) sheep tested positive and doubtful for anti‐H5 antibodies by ELISA, had MN titres of 1:20, but tested negative by both HI test and the anti‐NP antibody ELISA. Four sheep (#150, 152, 211 and 216) tested positive by the MN assay only (titres 1:20), while three others (#3, 65 and 120) tested inconclusive in the anti‐H5 antibody ELISA and negative by all other assays. Milk from 59 individuals, including sheep #68 and #76, all tested negative in both ELISAs and were not analysed further. Figure 2 shows ELISA results of individual sheep, with the HI‐positive individual highlighted in red. Data from individuals with positive results in one or more assays are provided in Table 1.

**TABLE 1:** Sheep testing positive in one or more assays for serum antibodies specific to type A influenza nucleoprotein (ELISA = ID screen Influenza A Antibody Competition Multispecies ELISA) or H5 subtype avian influenza (ELISA = ID screen Influenza H5 Antibody Competition 3.0 Multispecies ELISA, HI = haemagglutination inhibition test, MN = microneutralisation assay, Clade 2.3.4.4b = A/turkey/Italy/21VIR9520–3/2021 (H5N1), Euras LPAI (Eurasian low pathogenic avian influenza) = †A/teal/England/7394/2006 (H5N3) or ‡A/Mallard Duck/Netherlands/38/2008 (H5N3), § for sheep 68 only, additional HI tests with LPAI gull influenza A viruses (A/herring gull/Norway/10_2336/2006 (H13N6) and A/herring gull/Norway/10_1623/2006 (H16N3)) were performed and resulted negative; NVI = Norwegian Veterinary Institute, EURL = European Reference Laboratory for Avian Influenza and Newcastle disease, Istituto Zooprofilattico Sperimentale delle Venezie, np = not performed).

ID	Sex, year of birth	Detection of antibodies specific to						
		Type A influenza ELISA	H5 subtype avian influenza					
			ELISA	HI [NVI]		HI [EURL]		MN
		Result (S/N %)	Result (S/N %)	Result (titre)		Result (titre)		Result (titre)
				Clade 2.3.4.4b	Euras LPAI†	Clade 2.3.4.4b	Euras LPAI‡	Clade 2.3.4.4b
76	F 2022	+ (37,5)	+ (9,3)	+ (1:20)	—	+ (1:10)	—	+ (1:40)
68	F 2023	+ (34,9)	+/− (43,2)	—	—	—	—	—
140	F 2019	— (67,2)	+ (30,7)	—	—	—	—	+ (1:20)
10	F 2021	— (54,3)	+/− (42,7)	—	—	—	—	+ (1:20)
150	F 2021	— (82,8)	— (90,3)	np	np	—	—	+ (1:20)
152	F 2021	— (51,4)	— (67,2)	np	np	—	—	+ (1:20)
211	F 2023	— (84,5)	— (76,8)	np	np	—	—	+ (1:20)
216	F 2019	— (99,0)	— (80,3)	np	np	—	—	+ (1:20)
3	F 2023	— (89,3)	+/− (48,9)	—	—	—	—	—
65	F 2022	— (106,1)	+/− (48,8)	—	—	—	—	—
120	F 2018	— (86,9)	+/− (47,8)	—	—	—	—	—

## 3 Discussion

We here document the detection of antibodies specific to type A influenza and H5 subtype avian influenza across multiple assays in a single sheep in Norway. This was one of 220 sheep with documented exposure to substantial environmental contamination during a major HPAI outbreak in seabirds 11 months earlier, in 2023. Considering the degree of exposure, the young age of the sheep and the limited access to free‐range grazing in the period between the outbreak and sampling, it is likely that the antibody response originated from the seabird outbreak.

Detection of antibodies in one out of 220 sheep corresponds to a serological prevalence of approximately 0.5%. This may underestimate the true rate of spillover infections, as the 11‐month interval between exposure and sampling could have decreased the sensitivity of our surveillance. Six additional sheep showed partial serological evidence of H5 exposure, based on the detection of anti‐H5 antibodies in the ELISA and/or the MN assay, despite testing negative in the HI assay and for anti‐NP antibodies. Commercial ELISAs are not robustly validated for use in sheep, and we have limited knowledge of the diagnostic performance of the HI and MN assays in this species. Moreover, we only have a partial understanding of mammalian H5 avian influenza antibody response kinetics. The three assays used to detect anti‐H5 antibodies in our study differed in sensitivity. Positive results by HI test and H5 antibody ELISA (with a 40% cut‐off) were all confirmed by MN, suggesting either a higher sensitivity of the MN assay, as previously reported for humans [9], and/or a higher specificity of the HI and H5 ELISA. Antigenic variation is unlikely to have affected the assay sensitivity, as the outbreak H5N1 viruses belonged to the EA‐2022‐BB genotype [2] and differ from the virus used for HI and MN by only two HA1 positions (31 and 88), neither considered antigenically relevant. Interestingly, only one of the sheep with neutralising antibodies tested positive for the presence of anti‐NP antibodies, and another sheep tested positive for anti‐NP antibodies but showed no reactivity to H5, possibly reflecting variation in antibody responses to different viral proteins, although reactivity to other influenza A subtypes has not been ruled out. For comparison, cows infected with HPAIV H5N1 mount antibody responses to NP and H5 in both milk and serum within 1–2 weeks of infection [10], but the duration of each response remains undetermined. No antibodies were detected in milk in our study, suggesting that serum may be a more sensitive matrix than milk for retrospective serological HPAI surveillance in sheep.

Active HPAIV H5N1 infection was recently documented by Banyard et al. in a sheep in Great Britain, on a farm with an HPAI outbreak in captive birds [11]. Despite the absence of clinical signs, viral RNA was detected in milk, suggesting a similar tropism to that observed in cattle [3, 10]. In this sheep, HI titres peaked at 1:160, considerably higher than in our investigation. Antibody titres are strongly influenced by the time from the last exposure. In human patients with confirmed H5N1 infection, serum MN and/or HI titres 5 to 18 months post‐infection were reduced by eight‐fold or more compared to peak levels [12, 13]. If similar waning occurs in sheep, the HI titres detected in our investigation could be consistent with a peak response comparable to that observed by Banyard et al. [11]. Moreover, in their discussion of potential routes of infection, Banyard et al. suggest that infective material could have been introduced into the teat by suckling lambs. Our observations of lambs exploring diseased birds with their muzzles before feeding from their mothers support the relevance of this hypothesis, although no conclusions about entry point or infection site can be made.

As our study is based on retrospective serological investigations, we cannot exclude that the observed antibody response was triggered by prolonged mucosal exposure to high antigen loads in the absence of active infection. However, without viral replication, the induction of a humoral response typically requires mechanisms that allow the antigen to cross the epithelium, such as parenteral administration or the use of adjuvants [14]. Therefore, we propose that actual spillover infection remains the most plausible explanation for the detection of antibodies against both surface and internal viral proteins in a sheep with documented exposure to diseased birds in a highly contaminated environment. This indicates that HPAI spillovers from wild birds to sheep can occur given sufficient exposure, although the frequency appears to be low, even under conditions of high infection pressure.

HPAI in ruminants constitutes a zoonotic threat, with cattle identified as the likely source of 41 human cases during the ongoing US outbreak [5]. Our findings add to previous reports providing direct and indirect evidence of HPAI spillover from wild birds to small ruminants [11, 15]. From a One Health perspective, this underscores the need to prevent livestock contact with HPAI‐contaminated grassland, diseased birds and carcasses and to include small ruminants in HPAI outbreak investigation and surveillance.

## Author Contributions

**Johanna Hol Fosse:** conceptualization, investigation, funding acquisition, writing – original draft, methodology, visualization, writing – review and editing, formal analysis, supervision. **Grim Rømo:** conceptualization, investigation, writing – original draft, writing – review and editing, visualization, project administration. **Francesco Bonfante:** investigation, funding acquisition, writing – review and editing, validation, methodology, formal analysis, resources. **Ida Kristin Myhrvold:** methodology, writing – review and editing, investigation. **Kristin Stangeland Soetart:** investigation, writing – review and editing. **Kristin Udjus:** investigation, writing – review and editing, methodology. **Ragnhild Tønnessen:** conceptualization, funding acquisition, writing – original draft, project administration, writing – review and editing, investigation, supervision.

## Ethics Statement

Blood sampling was performed as part of post‐outbreak surveillance and falls within the definition “nonexperimental clinical veterinary practices”, which is not considered scientific research and hence does not require prior approval by the national animal research authority (DIRECTIVE 2010/63/EU).

## Consent

All participating farmers provided written informed consent for inclusion of their animals and related information in this publication.

## Conflicts of Interest

The authors declare no conflicts of interest.

## Supporting information


**Video S1:** Complementary to Figure 1. Footage showing lamb feeding from ewe immediately after close muzzle contact with a diseased seabird during the 2023 HPAI outbreak in northern Norway. Video by Grim Rømo.

## Data Availability

The full set of raw data from the study has been deposited in the diagnostic record system of the Norwegian Veterinary Institute, where they are stored in accordance with institutional and national data management protocols. Anonymised data can be provided upon request.
